# Anxiety and Mood Disorders on the Rise: Exploring Clinical Profiles and Risk Factors in the Netherlands

**DOI:** 10.1192/j.eurpsy.2025.271

**Published:** 2025-08-26

**Authors:** A. Gkitakou, M. Ten Have, N. M. Batelaan, A. Luik, B. W. Penninx

**Affiliations:** 1 Department of Psychiatry, Amsterdam UMC, Vrije Universiteit Amsterdam, Amsterdam Public Health Research Institute, Amsterdam; 2 Trimbos Institute, Netherlands Institute of Mental Health and Addiction, Utrecht; 3 Department of Epidemiology, Erasmus MC University Medical Center Rotterdam, Rotterdam, Netherlands

## Abstract

**Introduction:**

While an increase in mental disorders has been suggested, the role of societal changes, such as sociodemographic, vulnerability, or health-lifestyle factors, on this increase is scarce. This information is however crucial for health care policy and planning.

**Objectives:**

We examined trends in the 12-month prevalence of anxiety and mood disorders, their clinical profiles, and how sociodemographic, vulnerability, and health-lifestyle risk factors may have contributed to these trends.

**Methods:**

We used data from 11,615 respondents (mean age= 43.5, 53.5% female) of the Netherlands Mental Health Survey and Incidence Study, examining the general population in 2007-2009 (NEMESIS-2, n= 6,646) and 2019-2021 (NEMESIS-3, n= 4,969). The Composite International Diagnostic Interview 3.0 was used to determine DSM-IV diagnoses. Logistic regression and interaction analyses were then conducted to assess the association between risk factors and the prevalence of anxiety and mood disorders, while also evaluating changes over time between the two cohorts.

**Results:**

The 12-month prevalence of all types of anxiety and mood disorders significantly increased from 2007–2009 to 2019–2022, with increases ranging from 56% to 125%. Clinical profiles of those with disorders were not milder in 2019-2022; there was greater mental health care use, a higher number of disorders, and an earlier age of onset. There was no consistent evidence that sociodemographic, vulnerability, or health-lifestyle risk factors became more prevalent over time or had a greater relative impact over time.

**Conclusions:**

Our study showed a consistent increase in 12-month prevalence across all anxiety and mood disorders over the past decade. This rise in prevalence could not be explained by an increase in the absolute or relative impact of specific risk factors, nor were there significant differences in clinical profiles over time.

**Disclosure of Interest:**

None Declared

